# HOXA9 transcription factor is a double-edged sword: from development to cancer progression

**DOI:** 10.1007/s10555-023-10159-2

**Published:** 2023-12-08

**Authors:** U Sangeetha Shenoy, Divya Adiga, Faisal Alhedyan, Shama Prasada Kabekkodu, Raghu Radhakrishnan

**Affiliations:** 1 Department of Cell and Molecular Biology, Manipal School of Life Sciences, Manipal Academy of Higher Education, Manipal, Karnataka 576104 India; 2 Department of Oral Pathology, Manipal College of Dental Sciences, Manipal Academy of Higher Education, Manipal, 576104 India; 3 Department of Oral and Maxillofacial Surgery and Diagnostic Sciences, Prince Sattam bin Abdulaziz University, Al-Kharj, 11942 Saudi Arabia; 4 Unit of Oral and Maxillofacial Pathology, School of Clinical Dentistry, The University of Sheffield, Sheffield, United Kingdom

**Keywords:** HOXA9, Transcription factor, Molecular alterations, Cancer progression, Clinical application, Therapeutic targeting

## Abstract

The HOXA9 transcription factor serves as a molecular orchestrator in cancer stemness, epithelial-mesenchymal transition (EMT), metastasis, and generation of the tumor microenvironment in hematological and solid malignancies. However, the multiple modes of regulation, multifaceted functions, and context-dependent interactions responsible for the dual role of HOXA9 as an oncogene or tumor suppressor in cancer remain obscure. Hence, unravelling its molecular complexities, binding partners, and interacting signaling molecules enables us to comprehend HOXA9-mediated transcriptional programs and molecular crosstalk. However, it is imperative to understand its central role in fundamental biological processes such as embryogenesis, foetus implantation, hematopoiesis, endothelial cell proliferation, and tissue homeostasis before designing targeted therapies. Indeed, it presents an enormous challenge for clinicians to selectively target its oncogenic functions or restore tumor-suppressive role without altering normal cellular functions. In addition to its implications in cancer, the present review also focuses on the clinical applications of HOXA9 in recurrence and drug resistance, which may provide a broader understanding beyond oncology, open new avenues for clinicians for accurate diagnoses, and develop personalized treatment strategies. Furthermore, we have also discussed the existing therapeutic options and accompanying challenges in HOXA9-targeted therapies in different cancer types.

**Graphical Abstract:**

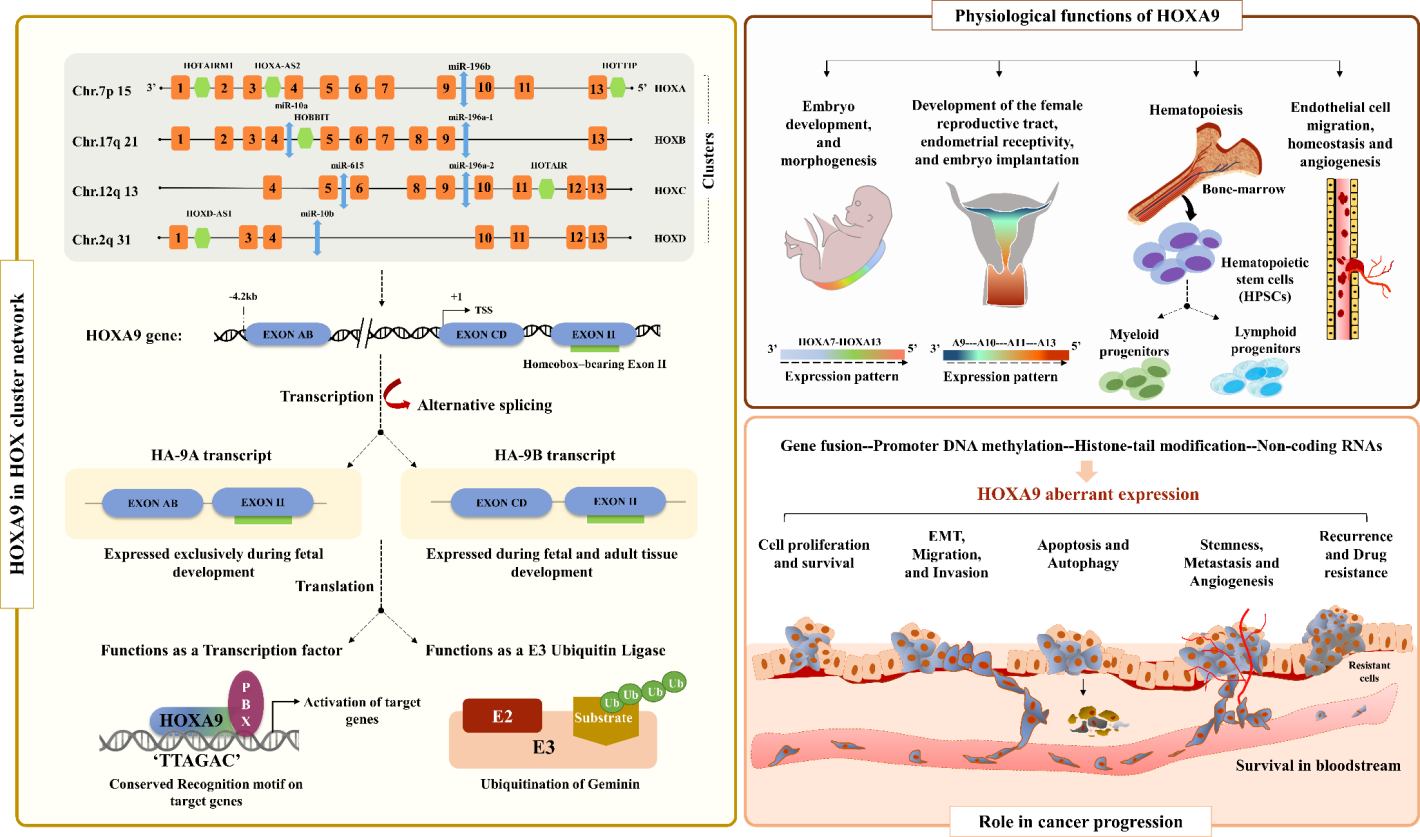

## Introduction

Homeobox (HOX) genes, originally discovered in *Drosophila melanogaster*, are evolutionally conserved genes formed as a result of successive duplication and subsequent divergence from primordial HOX cluster genes [1, 2]. In mammals, the 39 *HOX* genes are organized into 4 different HOX clusters, each located at different chromosomal coordinates [3]. Following translation, *HOX* genes primarily function as transcription factors with a key role in regulating signaling molecules [4]. These signaling molecules govern crucial biological events that are essential for maintaining normal physiology and tissue homeostasis. The expression pattern of *HOX* genes plays a pivotal role during embryogenesis in determining the body axis, skeletal morphology, and organ development in a tissue-specific manner. These transcription factors possess an intrinsic ability to bind to target genes through their DNA-binding homeodomain, thereby facilitating the activation of those genes. During embryogenesis, *HOX* genes are transcribed in a temporally and spatially collinear manner, wherein the position of each gene within the HOX cluster corresponds to sequential activation along the anterior-posterior axis [5]. This activation occurs from the 3’ end to the 5’ end of the HOX cluster, originating sequential developmental patterns [5–7].

The *HOXA9* gene has been extensively studied among the 39 *HOX* genes, particularly in the context of hematological as well as solid malignancies [8, 9]. Under normal physiological conditions, *HOXA9* has been recognized as a crucial regulator of various processes, including morphogenesis [10], embryo implantation [11], hematopoiesis [12], and endothelial cell regulation [13]. Numerous studies have revealed a wide range of genetic and epigenetic variations occurring at the *HOXA9* locus, shedding light on their biological implications during carcinogenesis. Genetic alterations, such as gene translocations and gene fusions at the *HOXA9* locus, have been observed to contribute to leukemic transformation in acute myeloid leukemia (AML) [14]. On the other hand, epigenetic factors play a role in the deregulation of *HOXA9* in various solid tumors. These include chromatin modification [15], promoter DNA methylation [16–18], gene-body methylation [19], microRNAs (miRNAs) [20–23], and long noncoding RNAs (lncRNAs) [24]. Aberrant expression of *HOXA9* triggers an impaired immune response, resulting in sustained activation of downstream signaling pathways. This disruption of normal physiology creates a microenvironment conducive to tumor growth.

This review aims to specifically explore the functional aspects of HOXA9 in normal physiologic conditions and its involvement in oncogenesis across various cancer types. Additionally, we have summarized the clinical significance of HOXA9, its role in drug resistance and recurrence, and important strategies to therapeutically target HOXA9 in cancer.

## Structural and genetic complexity of the ***HOXA9*** locus

Understanding the structural complexity of the *HOXA9* gene is crucial for studying its regulatory and functional roles. Throughout the evolutionary process, the 5’ end gene known as Abdominal B (AbdB) in *Drosophila* underwent duplication, resulting in the emergence of five AbdB-like genes positioned at the 5’ side of all four clusters in mammals. They exhibit similarities in the gene sequence and position in the HOX cluster; hence, each of the duplicated *HOX* genes can be aligned between the four clusters. Within the HOXA cluster, AbdB-like genes undergo duplication to form *HOXA9, HOXA10, HOXA11*, and *HOXA13* genes in mammals [25].

As a member of the HOXA cluster, *HOXA9* is located on the short arm of chromosome 7. Kim *et al*. sequenced the entire 7.2 kb sequence of the human *HOXA9* gene. They identified three different exons, of which exon AB and exon CD are located at the 5’ end, while exon II is located at the 3’ end [26]. Due to alternative splicing at exon CD, humans have two types of HOXA9-coding transcripts known as HA-9A and HA-9B. Both transcripts share a common 3’ exon II that contains the highly conserved ‘homeobox’ sequence for DNA binding. However, they differ in their 5’ exons. HA-9A proteins are exclusively present during foetal development. In contrast, HA-9B proteins, also known as canonical HOXA9, are found in both embryonic and adult tissues [26]. It is interesting to note that the *HOXA9* gene has numerous transcripts in various disorders. These transcripts are generated as a result of alternative promoter usage and alternative splicing of the *HOXA9* gene. Popovic et al. extensively reviewed the transcriptional complexity of the *HOXA9* gene, with particular emphasis on various human transcripts [27]. Alternative splicing at the exon CD splice site results in the generation of a premature stop codon prior to the homeobox region. This leads to the production of a protein lacking a homeodomain, which has been observed in both mice and humans [28, 29]. In contrast to the canonical isoform of *HOXA9*, this truncated isoform HOXA9T has been found both in the nucleus and cytoplasm of various tissues and is abundantly expressed in the embryonic genital tract, kidney, limb, and tail [27, 29]. The presence of a truncated isoform has been reported in endometrial adenocarcinoma cells. In these cells, both truncated isoforms and the canonical isoforms coexpress and compete for binding with several cofactors, including the CREB-binding protein (CBP) [29]. The truncated isoform interferes with the functions of the canonical isoform, causing molecular pathway disruption in various diseases [29]. However, a study on human MLL-rearranged leukemia demonstrated that both isoforms cooperate to promote leukemogenesis [30]. There is still much to be understood regarding the isoform-mediated molecular mechanisms involved in disease progression.

## Functional role of HOXA9 under normal physiological conditions

### Embryo development and morphogenesis

*HOX* genes are well known for their role in determining anterior-posterior segmental identity, contributing to the formation of skeletal structures, and playing a key role in organogenesis [10, 31]. In mammals, *HOXA9* is involved in the morphogenesis of the embryonic skeletal system and the specification of the posterior part of the embryo [10]. The *HOXA9* and *HOXD9* paralogous genes are specifically involved in the axial skeletal patterning of the lumbosacral region and forelimb morphogenesis at the stylopodal stage. This was demonstrated by developing HOXA9^-^/HOXD9^-^ double mutants in mice [32]. Mutations in all HOXA cluster genes can lead to the development of defects in the cardiac, respiratory, and urinogenital systems [31]. Frameshift mutations in six *HOX* genes, namely, *HOXA9, HOXA10, HOXA11, HOXD9, HOXD10*, and *HOXD11* have been associated with severe limb malformation. Moreover, HOXA*9,10,11* negative-mutant mice displayed a reduced length of the ulna and radius, implying their major role in stylopod development [33].

### Female reproductive tract development, endometrial receptivity, and embryo implantation

The posterior or 5’ end *HOX* genes (*HOXA9 to HOXA13*), belonging to the AbdB family, are involved in the development of the mammalian female reproductive system [34]. During embryonic development, 5’ *HOX* genes, including *HOXA9*, exhibit robust expression in the paramesonephric duct. During postnatal differentiation, these follow a spatial axis, indicating their specific patterns of expression along the developing tissues or organs. *HOXA* cluster genes actively govern crucial physiological processes in the female reproductive tract throughout the adult stage [34, 35]. It is interesting to note that *HOXA9* is expressed in the fallopian tube. Hence, aberrant expression of *HOX* genes leads to developmental abnormalities in the uterus, impaired growth of the endometrium, and a reduced implantation rate [34, 36]. Ovarian steroids such as progesterone and estrogen have the ability to induce the activation of *HOXA9*, *HOXA10*, and *HOXA11* in a collinear manner [37]. Hormonal regulation of these *HOX* genes indicates the functional interaction between HOX factors and hormonal nuclear receptor families during implantation [37–40]. Considering the conserved sequences and overlapping expression pattern similar to *HOXA10*, Xu *et al*. extensively studied the role of *HOXA9* and other *HOX* genes that are highly expressed in the endometrium during menstruation, and their expression is correlated with implantation time [11]. Interestingly, they found a significant reduction in the implantation rate upon shRNA-mediated knockdown of maternal *HOX* genes in mouse models, indicating that *HOX* genes are also essential for endometrial receptivity [11].

### Hematopoiesis

Over the past decades, studies have deciphered the role of *HOX* genes in hematopoiesis [41]. The mammalian *HOXA9* gene plays a remarkable role in hematopoiesis, and its expression is closely associated with blood formation [41, 42]. *HOXA9* is abundantly expressed in hematopoietic stem and progenitor cells (HSPCs), and its expression diminishes upon differentiation [43, 44]. Researchers investigated the deleterious influence of *HOXA9* on primitive blood cells by developing HOXA9^-^/^-^ mutant mice. Interestingly, the mice showed a significant reduction (30–40%) in the leukocyte and lymphocyte populations (P value < 0.05). In addition, they not only observed a reduction in the myeloid and erythroid progenitors but also witnessed the reduced size of the spleen and thymus [45]. A group of researchers have demonstrated that the binding of CDX4 followed by Menin-dependent H3K4 trimethylation could induce *HOXA9* expression during normal hematopoiesis [12].

Initially, hemogenic precursors (HEPs) (CD31^+^CD34^+^CD45^−^) show the highest expression of *HOXA9*, and the expression level is reduced when HEPs undergo differentiation into CD45^+^ blood cells [46]. In other words, in vivo overexpression and knockdown studies have revealed that *HOXA9* plays a major role in the differentiation of human embryonic stem cells (hESCs) by facilitating the transition of HEPs into CD45^+^ blood cells by regulating the NF-κB pathway [46]. A recent study revealed that *HOXA9* overexpression specifically enhances myeloid potential, rather than erythroid potential, by promoting cell cycle progression through the upregulation of the NF-κB pathway [47]. Researchers have performed ChIP sequencing to investigate the regulation and mechanisms behind the role of *HOXA9* in hematopoiesis and leukemia. The study made an intriguing observation that HOXA9 proteins in conjunction with cofactors form associations with enhanceosomes at several highly conserved target sites. This interaction plays a crucial role in promoting stem cell expansion and B/T-cell development. The gene expression profiling data indicated that *HOXA9* either promotes or suppresses target genes based on the type of cell and chromatin content. Various upstream oncogenic alterations lead to persistent activation of *HOXA9*. This activated form of HOXA9 binds with MEIS1 and several other lineage-restricted transcription factors, enabling the recruitment of P300/CBP to transcriptionally activate the target genes involved in proliferation and leukemic transformation [48].

### Endothelial cell proliferation, homeostasis, and angiogenesis

Endothelial cell (EC) migration is an important phase in vascular system development. On the other hand, ECs are formed during embryogenesis through the differentiation of angioblasts, a process known as vasculogenesis. This process ultimately leads to the development of new blood vessels [49]. However, angiogenesis is the process through which newly generated blood vessels differentiate to become a complex network of new blood vessels in adulthood. Vasculogenesis and angiogenesis are crucial for maintaining vascular integrity [50]. Endothelial cell migration is needed in angiogenesis and wound healing [50]. The HOXA9-mediated activation of signaling pathways involved in this process is still under investigation [51]. Patel et al. were the first to identify HOXA9 as the sole member of the HOX family to be expressed in EC. They identified a novel splice variant, HOXA9EC, screening a human cDNA library. HOXA9EC is regulated by a novel promoter and is exclusively expressed in EC. This splice variant was found to be sensitive to TNF-α, and its expression was downregulated upon TNF-α activation. This suggests that the new promoter is activated by cytokines and contributes to maintaining ECs in a quiescent state [13]. Hence, during cytokine activation, the HOXA9 transcription factor plays a role in modulating essential genes associated with EC activation [13]. During inflammation, the release of TNF-α induces E-selectin activation. E-selectin is a leukocyte adhesion molecule that enables the recruitment of leukocytes and firm adhesion of leukocytes to inflammatory foci [52]. This cytokine-mediated activation is facilitated by the binding of NF-κB to the E-selectin promoter region [53]. Later, the same group of researchers found that the constituent expression of *HOXA9* prevents EC activation by inhibiting NF-κB-mediated transcriptional activation of E-selectin in human umbilical vein endothelial cells (HUVECs) [54, 55].

In a later study, Zhang et al.,2012 demonstrated the potential of HOXA9EC in reducing endothelial dysfunction induced by high glucose [56]. This leads to the downregulation of crucial endothelial genes such as eNOS (endothelial nitric oxide synthase), VEGFR2 (vascular endothelial growth factor receptor 2), and VE-cadherin. Furthermore, the overexpression of HOXA9EC could serve as a viable strategy to mitigate endothelial dysfunction [56].

It is interesting to note that HOXA9 is crucial for endothelial cell migration and tube formation. This was demonstrated through siRNA-mediated transfection of HOXA9 into HUVECs. The study elaborated on the proangiogenic nature of HOXA9 and revealed that EphB4 receptor tyrosine kinase is a novel downstream target of HOXA9. Depletion of HOXA9 resulted in the downregulation of EphB4 expression, which in turn led to reduced migration and impaired tube formation in endothelial cells [57, 58]. Additionally, the ChIP assay provided evidence that HOXA9 directly interacts with the EphB4 promoter, thereby facilitating its transcriptional activation. Furthermore, the study also showed that HOXA9 stimulates EphB4 by binding to its TAAT motif located − 1365 bp upstream of the TSS in the promoter region. This was confirmed through site-directed mutagenesis and deletion constructs [58].

## Regulation of HOXA9 in cancer

The structural complexity of *HOXA9* plays a significant role in regulating multiple aspects of cancer. Notably, the regulation of *HOXA9* in different cancer types is highly context-dependent. Surprisingly, the mode of regulation can vary not only between cancer types but also within subtypes of the same malignancy. Previous reports have indicated that HOXA9 can be regulated by both genetic and epigenetic factors in various types of cancer.

*HOXA9* is mainly modulated through genetic fusion in leukemia. The chromosomal translocation t (7; 11) (p15; p15) results in the fusion of HOXA9 with nucleoporin 98 kDa (NUP98), leading to the formation of a chimeric protein called NUP98-HOXA9 (NHA9), which is observed in various malignancies [59]. The study used oligonucleotide microarray analysis to investigate the effects of the NUP98-HOXA9 fusion on hematopoietic cell proliferation, differentiation, and leukemic transformation [14]. ChIP-seq analysis revealed that MLL1 occupancy with the NHA9 protein is crucial for the activation of downstream target genes involved in leukemogenesis [60, 61]. Amplification of *HOXA9* was evident in angiosarcoma, contributing significantly to abnormal vascular cell proliferation and blood vessel growth [62]. In addition to gene fusions, *HOXA9* has also been regulated in multiple ways by means of epigenetic factors in AML. Aryal et al. performed an extensive literature search and summarized the molecular regulators of HOXA9 in AML. The study also outlined the different kinds of drugs that were used to target the oncogenic nature of HOXA9 in AML [63].

*HOXA9* is subjected to complex regulation involving multiple epigenetic factors in certain cancer types. It is epigenetically regulated by promoter DNA methylation, miRNAs, lncRNAs, and histone complexes in different malignancies (Table 1).

**Table 1:** Epigenetic regulation of HOXA9 in cancer

Cancer	Expression of HOXA9	Molecular mechanism	Clinical significance	References
**HOXA9 is regulated by promoter DNA methylation**				
Lung adenocarcinoma	↓	HOXA9 methylation, High frequency of TP53 mutations, and the upregulation of the OncomiR miR-9	Associated with poor prognosis	[116]
Non-Small Cell Lung Cancer	↓	Transcriptional downregulation of HOXA9	Associated with poor recurrence-free survival	[107, 163]
High-Grade Non-Invasive Bladder Cancer	↓	Increased methylation in recurrent tissues	Associated with recurrence and disease-specific mortality	[109]
Head and neck squamous cell carcinoma	-	HOXA9 promoter hypermethylation	Correlates with metastasis and advanced tumor stage	[17, 137]
**HOXA9 is regulated by miRNAs**				
Non-Small Cell Lung Cancer	↓	miR-182-5p sponges HOXA9; miR-196b sponges HOXA9	Contributes to tumor initiation and progression; Promotes invasiveness	[66, 164]
Acute myeloid leukaemia	↑	miR-182/miR-196b downregulation; Reduced sponging effect of miR-182/ miR-196b/ with HOXA9	Promotes leukemogenesis	[165, 166]
Oral squamous cell carcinoma	↑	miR-139-5p downregulation; Reduced sponging effect of miR-139-5p with HOXA9	Promotes proliferation, invasiveness, and migration	[167]
Osteosarcoma	↑	miR-1294 downregulation; Reduced sponging effect of miR-1294 with HOXA9	Promotes proliferation and invasion	[21]
Epithelial ovarian cancer	↓	Enhanced sponging effect of miR-196b on HOXA9	Promotes ovarian cancer cell invasiveness; Associated with recurrence	[22]
Colorectal cancer	↑	miR-133b downregulation; Reduced sponging effect of miR-133b with HOXA9	Promotes cancer metastasis	[103]
Glioma	↑	miR-647/miR-638 downregulation; Reduced sponging effect with HOXA9	Promotes cell proliferation, colony formation and invasion	[67, 68]
Uveal melanoma	↓	Enhanced sponging effect of miR-652 on HOXA9; Promoted HIF-1α signaling	Promotes cell growth and migration	[168]
**HOXA9 is regulated by other ncRNAs**				
Prostate cancer	↑	TWIST1/WDR5/HOTTIP induces HOXA9 expression	Promotes migration, invasion, and metastasis	[104]
Pancreatic cancer	↑	LncRNA HOTTIP induces HOXA9 expression	Promotes proliferation and migration	[169]
Triple-negative breast cancer	↓	LncRNA MIR503HG regulates miR-224-5p/HOXA9 axis	Promotes proliferation, migration, and invasion	[24]
Colorectal adenocarcinoma	↑	LncRNA PCED1B-AS1 regulates miR-633/HOXA9 axis	Promotes cell proliferation and reduces apoptosis	[170]
Gastric cancer	↑	CirRNA circ_0026359 regulates miR-140-3p/HOXA9 axis	Associated with poor prognosis	[100, 171]
Osteosarcoma	↑	LncRNA DLX6-AS1 regulates miR-641/HOXA9 axis	Promotes proliferation, migration, and invasion	[172]
Laryngeal squamous cell carcinoma	↑	LncRNA KCNQ1OT1 directly binds with HOXA9	Promotes proliferation, invasion, and metastasis	[173]
**HOXA9 is regulated by histone-complexes**				
Breast cancer	↓	TET1 demethylates HOXA9 promoter	Promotes tumor growth and metastasis	[99]
Acute myeloid leukaemia	↑	G9a methyl transferase promotes HOXA9 transcription	Promotes expansion and differentiation of AML cells	[174]

*Note*: ↑- Upregulated; ↓-Downregulated

Indeed, *HOXA9* has been found to be regulated by several miRNAs during hematopoiesis and cell differentiation [64, 65]. Deregulation of upstream regulators frequently results in abnormal expression of *HOXA9*, resulting in tumor aggressiveness. *HOXA9* is regulated by various miRNAs in solid cancers (Table 1), including miR-196b, which is embedded within the HOX cluster [22, 66]. Furthermore, overexpression of *HOXA9* in glioma is mainly due to the downregulation of miR-638 and miR-647. The reduced sponging effect on *HOXA9* triggers its oncogenic potential, causing enhanced cell proliferation, colony formation, and invasion [67, 68]. In addition, evidence supports that other ncRNAs, such as lncRNAs and circRNAs, are often involved in the molecular sponging mechanism to regulate *HOXA9* in cancer (Table 1).

Downregulation of *HOXA9* due to promoter-DNA methylation serves as a tumor suppressor and prognostic marker in solid tumors [9]. In addition to promoter DNA methylation, increased methylation at the first exon of *HOXA9* has also been correlated with gene repression in cervical cancer (CC). Restoring the expression of *HOXA9* in CC cell lines resulted in reduced cell proliferation and migration [19]. Remarkably, a unique interaction exists between *HOXA9* and *HOXA10* promoters in breast cancer (BC). The CpGs of *HOXA10* function as enhancers of the *HOXA9* gene, causing long-range chromatin interactions [18].

Understanding the precise processes underpinning *HOXA9* regulation in different cancer types is critical for designing targeted therapeutics and improving patient outcomes.

### Functional implications of HOXA9 in cancer progression

**Fig. 1:**
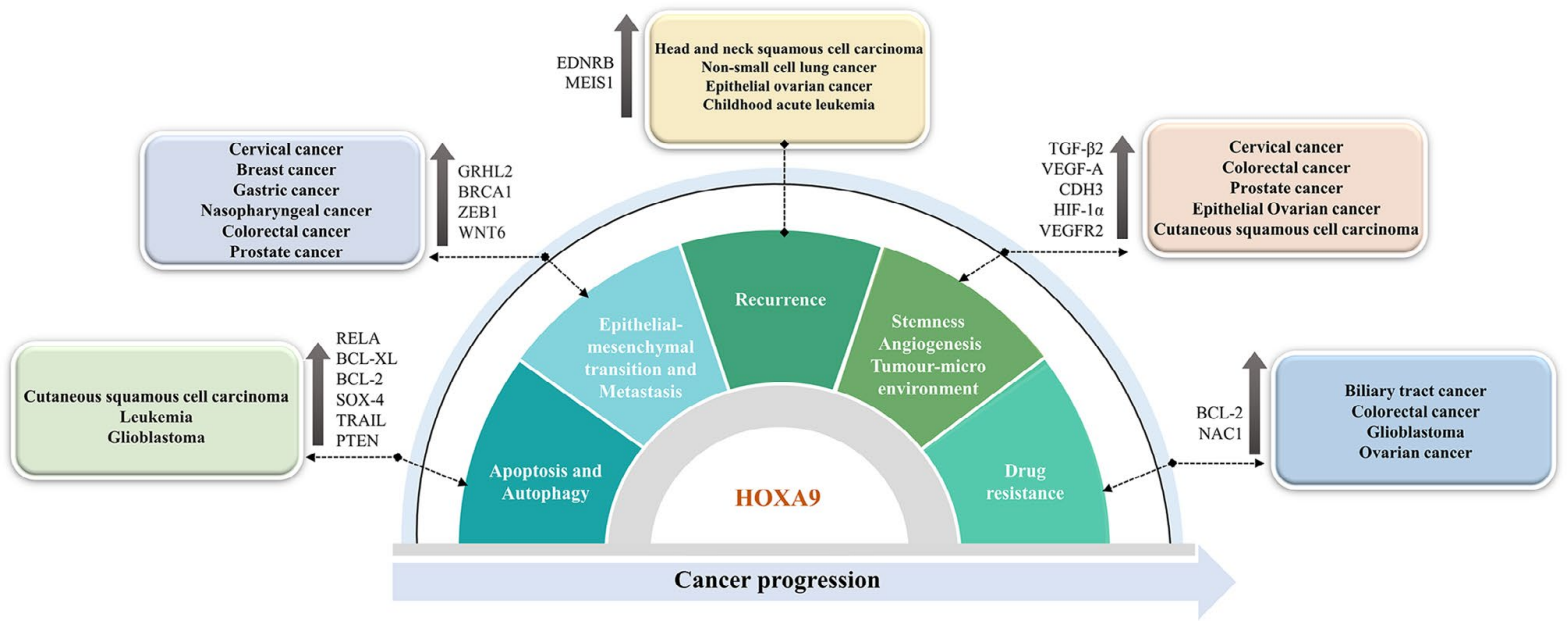
Role of aberrantly expressed HOXA9 in the acquisition of cancer hallmarks in different malignancies

Deregulation of *HOXA9* has been extensively studied in hematological as well as solid malignancies, including gastrointestinal cancers, skin-related cancers, head and neck cancer types, and gynecological cancers [69, 70] (Fig. 1). However, the role of *HOXA9* as an oncogene or tumor suppressor gene particularly depends on tumor heterogeneity. Therefore, it is crucial to investigate the molecular mechanisms underlying the regulation of HOXA9 and its implication in cancer progression. This section will discuss HOXA9-mediated cancer-associated molecular events in detail, which could provide valuable insights for the design and development of targeted therapies for cancer.

#### HOXA9 in the tumor microenvironment, stemness, and angiogenesis

The dynamics of the tumor microenvironment (TME) exhibit the characteristics that encompass tumor growth, angiogenesis, invasion, and metastatic dissemination. The niche around the tumor mass often consists of a variety of cell types, such as inflammatory immune cells, adipocytes, endothelial cells, and fibroblasts, in the stroma, which conjugate with cancer cells to release signals that favor the migration and invasiveness of the tumor mass and thus help to establish the TME [71, 72]. In other words, aggressive growth is triggered by the growth factors and chemokines secreted by the inflammatory cells of the TME, which helps to gain cancer stemness and sustain cancer hallmarks, which in turn leads to the transition into distant metastasis [71]. Once the TME is generated, the hypoxic environment often induces the formation of abnormal vasculature favorable for the growth of cancer cells [73]. Hence, there always exists an intricate connection between the TME, angiogenesis, and stemness.

Researchers have deciphered the crosstalk between HOX transcription factors and the TME in PCa [74]. Another study on PCa demonstrated that the *HOXA2, HOXA9*, and *HOXA10* genes play a crucial role in promoting tumor growth and progression. Aberrantly expressed HOXA2, HOXA9, and HOXA10 were found to be involved in the recruitment and infiltration of immune cells, including dendritic cells, macrophages, and mastocytes, during the progression of PCa [75]. In contrast, HOXA9 was found to be involved in the modulation of the NF-κB pathway, particularly during viral infection in CC. The study discovered a novel isoform of HOXA9 that lacks the homeodomain and is highly expressed in nontumorigenic HaCaT cells compared to CC cell lines and tumor samples. Restoring HOXA9 expression in CC cell lines resulted in the elevated expression of antigen-presenting cells (APCs) and regulators of the immune response, including IL17RD, FADD, CHEK2, TRAIL, IL-3/IL-5 pathways, and IFN-gamma signaling molecules [76].

Ko et al. in 2012 reported that elevated expression of HOXA9 in epithelial ovarian cancer (EOC) contributes to angiogenesis and the generation of a microenvironment for tumor growth [77]. This is due to the HOXA9-mediated transcriptional activation of *TGF-β2*, followed by subsequent activation of *CXCL12, IL-6*, and *VEGF-A* in peritoneal fibroblasts. Hence, HOXA9 promotes peritoneal fibroblasts and mesenchymal stem cells (MSCs) to acquire the properties of cancer-associated fibroblasts (CAFs) [78]. Through in vitro knockdown studies, the study also proved that the tumor-inducing property of HOXA9 is mediated only through the induction of *TGF-β2* in EOC cells. HOXA9 further induces the immunosuppressive phenotype of peritoneal macrophages by inducing the expression of *TGF-β2* and chemokine (C-C motif) ligand 2 [79]. Once a suitable environment is generated, floating cancer cells tend to implant in peritoneal mesothelial cells by means of the cell adhesion molecule P-cadherin to form aggregates and avoid anoikis [80]. HOXA9 induces the expression of the target gene *CDH3*, which encodes P-cadherin, enabling ovarian cancer (OVC) cells to adapt to the peritoneal environment and acquire an aggressive phenotype [81].

Furthermore, the TME often consists of cancer stem cells (CSCs) that possess self-renewal, differentiation, and treatment-resistance capabilities [82]. Targeting CSCs is essential to prevent recurrence, overcome therapy resistance, and inhibit metastasis. Interestingly, HOXA9 is overexpressed in colorectal cancer (CRC) patients with lymph node metastasis, and it contributes to stem cell overpopulation, which could favor cancer progression [83, 84]. Indeed, it was demonstrated that *HOXA9* is regulated by retinoic acid (RA) signaling, and an anticancer effect is exerted with all-trans retinoic acid (ATRA) treatment through the repression of *HOXA9* in CRC [83, 85].

In addition, a study on cutaneous squamous cell carcinoma (CSCC) reported that HOXA9 plays a crucial role in reducing the hypoxic response, angiogenesis, and tumor progression. Under normal physiological conditions, HOXA9 epigenetically regulates the key factor for the hypoxia response, HIF-1α. However, during CSCC tumor progression, miR-365 inhibits *HOXA9* expression, leading to the upregulation of HIF-1α and downstream genes (*GLUT1*, *HK2*, and *PDK1)* involved in the glycolytic pathway. In cells expressing HOXA9, it exerts a tumor-suppressive role by restricting the uptake of glucose and suppressing glycolysis in cancer cells. Therefore, the miR-365-HOXA9-HIF-1α regulatory axis could serve as a suitable target for intervention therapy [86, 87]. Furthermore, HOXA9 has been considered a master switch that directly regulates endothelial-committed target genes such as eNOs (endothelial nitric oxide synthase), *CDH5* (VE-cadherin), and *VEGFR2* to maintain the vasculature and angiogenesis [88]. The study also revealed that HOXA9-deficient mice exhibited impaired postnatal neovascularization [74, 88]. Hence, the expression of HOXA9 in endothelial progenitor cells is crucial for the maintenance of the tumor microenvironment and for the commitment to the endothelial cell lineage.

#### HOXA9 in the cell cycle, apoptosis, and autophagy

HOX transcription factors play a critical role in regulating cell proliferation during embryogenesis and morphogenesis. HOX transcription factors regulate the cell cycle machinery through protein‒protein interactions. Dysregulation of HOX transcription factors disrupts the cell cycle machinery, leading to cancer progression [89].

HOXA9 was identified as a potential regulator of the apoptosis and autophagy pathways in CSCC through the transcriptional control of RELA, a crucial element of the NF-κB pathway. In cells depleted of HOXA9, RELA was upregulated, leading to transcriptional activation of the antiapoptotic factor *Bcl-XL* and the autophagic molecules *ATG2, ATG3*, and *ATG12*. Accordingly, the study revealed that regulation of the NF-κB pathway by HOXA9 resulted in increased apoptosis and decreased autophagy in CSCC [90]. However, persistent activation of HOXA9 is needed for cell proliferation and survival. In leukemia, HOXA9-mediated maintenance of *BCL2* expression is essential for HOXA9-dependent immortalization of hematopoietic cells and survival of myeloid progenitor cells. Further research is warranted to elucidate the molecular interactions between HOXA9 and Bcl-2 family members to develop effective therapeutic options [91]. Recently, Miyamoto et al. showed that HOXA9 plays a multifunctional role in regulating antiapoptotic pathways in leukemia. The activation of HOXA9 target genes such as *BCL2* and *SOX4* was attributed to the combined activation of *MYC* and *HOXA9* caused by MLL fusion. The enhanced activation of these molecules not only repressed apoptosis but also promoted leukemogenesis, indicating that HOXA9 could be a potential regulator of apoptotic pathways [92].

Researchers have identified transcriptional cooperation between HOXA9 and JAK3/STAT5 in T-cell acute lymphoblastic leukaemia [93]. Furthermore, HOXA9 acts as a transcription factor for the oncogenic kinase enzyme PIM1, leading to the transcriptional activation of PIM1. The binding of HOXA9 to PIM1 facilitates the phosphorylation of the pro-apoptotic protein BAD, resulting in its inactivation. Hence, HOXA9-mediated activation of PIM1 kinase often results in enhanced proliferation and anti-apoptosis in leukaemia [94]. In glioblastoma (GBM), HOXA9 inhibits apoptosis by regulating tumor necrosis factor-related apoptosis-including ligand (TRAIL). The study also showed that increasing HOXA9 levels influence PTEN, a PI3K pathway antagonist, which in turn influences proliferation and apoptosis. As a result, reversing HOXA9 activation via PI3K inhibition may have therapeutic implications in human GBM [95].

#### HOXA9 in EMT and metastasis

HOXA9 demonstrates a dual role as a tumor suppressor and oncogene, with its function being context-dependent (Table 2). The expression of HOXA9 was found to be reduced in CC, and its restoration resulted in decreased cell proliferation and migration along with an increase in epithelial phenotype [19]. HOXA9 elevation led to a significant upregulation of the novel suppressor of EMT, GRHL2 (Grainyhead-like 2), compared to the scrambled control [19, 96, 97]. The study showed that CC cells expressing HOXA9 and infected with E6 or E7-HPV 18 oncoproteins demonstrated decreased cell proliferation, motility, colony formation, and metabolism. In fact, HOXA9 was found to be upregulated in E6- and E7-depleted cells [19]. Therefore, finding the molecular link in this phenomenon paves the way to inhibit CC progression.

**Table 2:** Oncogenic and tumor suppressive function of HOXA9 in cancer

Cancer	Functional implications upon aberrant expression	References
**HOXA9 as an oncogene**		
Colorectal cancer	Contributes to stem cell overpopulation,	[83, 84, 103]
Pancreatic cancer	Enhances the stem cell properties	[175]
Ovarian cancer	Contributes to the angiogenesis and generation of a microenvironment; Induces aggressive phenotype	[77, 81]
Leukemia	Promotes cell proliferation and survival; Reduces apoptosis	[92, 165]
Gastric cancer	Associated with tumor-node-metastasis (TNM) staging	[100]
Nasopharyngeal cancer	Associated with tumor-node-metastasis (TNM) staging	[101]
Prostate cancer	Induction of metastatic phenotype	[104]
Osteosarcoma	Promotes proliferation and invasion	[21]
Oral squamous cell carcinoma	Promotes proliferation, invasiveness, and migration	[167]
Glioma	Promotes cell proliferation, colony formation and invasion; Inhibits apoptosis	[67, 95, 105]
**HOXA9 as a tumor suppressive gene**		
Cutaneous squamous cell carcinoma	Downregulation of HOXA9 induces hypoxic response, angiogenesis, and tumor progression	[87, 90]
Cervical cancer	Downregulation of HOXA9 induces cell proliferation, migration and EMT	[19]
Breast cancer	Reduced levels of HOXA9 induces metastasis and aggressiveness	[98, 99]
Non-small cell lung cancer	Downregulation is correlated with disease recurrence; Promotes invasiveness	[66, 107, 163, 164]
High-Grade Non-Invasive Bladder Cancer	Promotes recurrence	[109]
Uveal melanoma	Promotes cell growth and migration	[168]
Lung adenocarcinoma	Associated with poor prognosis	[116]

Reduced levels of *HOXA9* transcripts have been associated with metastasis and aggressiveness in breast cancer (BC). The tumor suppressor gene *BRCA1* was expressed more frequently after HOXA9 restoration, which in turn prevented the malignant behavior of BC cells [98]. In addition, researchers have performed an extensive study on BC and found that inhibiting the HMGA2-TET1-HOXA9 pathway aided tumor development, intravasation, invasion, and metastasis in BC [99]. In contrast, HOXA9 was significantly upregulated in colonic adenoma, gastric cancer (GC), nasopharyngeal cancer (NPC) and CRC and was associated with tumor-node-metastasis (TNM) staging and positive lymph node metastasis [84, 100–102]. Especially in CRC, *HOXA9* upregulation was the consequence of miR-133b downregulation. Interestingly, the study found that elevated levels of *HOXA9* significantly contribute to tumor invasion and metastasis by regulating the expression of *ZEB1*. Restoration of miR-133b and downregulation of HOXA9 significantly reduced the expression of *ZEB1* and increased the expression of *CDH1* in CRC cells. Hence, this molecular pathway therefore serves as a possible therapeutic target for CRC treatment [103]. In 2017, Malek et al. extensively investigated the molecular mechanism of PCa metastasis and aggressiveness. A major EMT transcription factor, TWIST1, induces *HOXA9* expression either by transcriptional activation or by epigenetic reprogramming of the *HOXA9* locus. TWIST1 binds with a complex of proteins associated with SET1 (COMPASS)-like complex to induce chromatin modification at the *HOXA9* promoter via H3K4me3 in PCa cells. The induction of the metastatic phenotype was due to HOXA9 overactivation caused by the binding of the TWIST1-WDR5 complex to the E-box consensus sequence of the *HOXA9* promoter. The study demonstrated that pharmacological inhibition of HOXA9 using 10 nM HXR9 peptide effectively reduced PCa cell migration and invasion and prevented metastasis induced by TWIST1-HOXA9 [104].

Upregulation of WNT6, a crucial regulator of the Wnt/β-catenin pathway, was associated with poor clinical outcomes in glioma patients. By performing in vitro and in vivo studies, researchers found that glioma aggressiveness might be due to HOXA9/WNT6-mediated activation of the canonical Wnt/β-catenin pathway. There exists a molecular link between HOXA9 and WNT6 where the binding of HOXA9 to the promoter region of *WNT6* induces its expression. Thus, targeting the WNT6-HOXA9 pathway may be a promising therapeutic approach to inhibit the growth of glioma [105].

#### HOXA9 in recurrence

Although there have been considerable advances in therapeutic regimens, the recurrence rate of many types of cancer is still very high. Contemplating HOXA9 as a clinical biomarker not only facilitates the assessment of disease recurrence and cancer progression but also helps in treatment decisions in recurrent cancer patients. Interestingly, few recent studies have shown the role of *HOXA9* in recurrence in different cancer types. In head and neck squamous cell carcinoma (HNSCC), the study showed a correlation between *EDNRB* and *HOXA9* methylation in surgical margin imprints. Multivariate analysis revealed that the regions with a high risk of locoregional recurrence showed higher methylation upon surgery and hence were considered valuable predictive biomarkers for locoregional recurrence in HNSCC (HR, 3.31; P value = 0.012) [106].

According to a study on non-small cell lung carcinoma (NSCLC), *HOXA9* may be a useful marker for anticipating disease recurrence. Approximately 70% of NSCLC patients had *HOXA9* promoter hypermethylation and downregulation, which was associated with poor recurrence-free survival (P value = 0.01) [107].

A study on nonmuscle invasive bladder cancer (NMIBC) reported that hypermethylation status of *HOXA9*, *ISL1* (ISL LIM homeobox 1), and *ALDH1A3* (aldehyde dehydrogenase 1 family, member A3) and decreased expression of these genes were associated with aggressive clinical characteristics [108]. In a similar vein, Kitchen et al. in 2015 revealed that *ISL1/HOXA9* methylation might be used as a predictive biomarker for tumor recurrence in high-grade noninvasive bladder cancer within a year of cancer diagnosis. Indeed, *ISL1/HOXA9* gene methylation levels were greater in progressive and recurrent tumors than in nonrecurrent tumors (P value < 0.05), and these methylation levels were also related to disease-specific mortality [109]. Especially in patient urine samples of bladder cancer, the study showed that methylation levels of five markers *(HOXA9, POU4F2, TWIST1, VIM*, and *ZNF154*) were significantly linked to tumor recurrence. Univariate cox-regression analysis proposed these as markers for the early detection of recurrence in bladder cancer [110].

In contrast, HOXA9 and its co-factor MEIS1 upregulation were inversely correlated with relapse in pediatric acute leukemia [111]. In addition to tissue samples, researchers have investigated the prognostic significance of the methylation status of *HOXA9* in circulating tumor-specific DNA (ctDNA) in the blood samples of EOC patients during chemotherapy [112–114]. Studies have shown that *HOXA9* methylation could be used as a potential ctDNA biomarker to predict prognosis and recurrence and assess treatment resistance in different cancer types [112, 113, 115, 116]. Cai et al. conducted a comprehensive meta-analysis and confirmed that *HOXA9* methylation has potential as a reliable biomarker for predicting the prognosis of malignant tumors [9]. Interestingly, the study observed that the increase in methylation of *HOXA9* in patient blood samples after one treatment cycle was associated with reduced overall survival, especially in patients with disease recurrence [113].

#### HOXA9 in drug resistance

Despite advanced clinical approaches to target cancer cells, they still undergo metastasis and acquire an aggressive phenotype. The treatment ineffectiveness is due to the ability of cancer cells to resist the treatment [117]. Therapeutic regimens are typically designed to promote the immune response against growing tumors. This may involve administering specific drugs at the maximum tolerated dose (MTD) [118, 119]. While this method of treatment initially helps in depleting cancer cells, it often leads to impaired immune surveillance and drug resistance over time [120, 121]. Drug resistance is acquired due to the dynamic interaction between the host immune system and cancer cells. Initially, cancer cells respond effectively to therapies, but eventually, they tend to reoccur over a period of time [121].

A study on BRCA-mutated OVC has indicated that *HOXA9* promoter methylation in the plasma of patients could serve as a promising prognostic biomarker. *HOXA9* methylation in ctDNA was associated with the worst outcomes in PARP inhibitor-treated, platinum-resistant BRCA-mutated OVC. Patients with higher HOXA9 methylation in ctDNA had shorter median progression-free survival (PFS) than patients with lower methylation levels (P value < 0.05) after three treatment cycles [112].

The clinical significance of *HOXA9* methylation was investigated in patients with biliary tract cancer (BTC). The patients receiving erlotinib and bevacizumab drug treatment showed an increase in *HOXA9* methylation levels, which negatively correlated with patient survival [122]. In contrast, researchers have suggested that chemotherapy resistance in high-grade serous ovarian cancer (HGSOC) samples is due to the upregulation of HOXA9 [123]. In CRC, nucleus accumbens‑associated protein 1 (NAC1) contributes to drug resistance by inducing the expression of HOXA9 [124]. Researchers conducted a comprehensive study on GBM and identified HOXA9 as a key factor in promoting cancer stemness, aggressiveness, and drug resistance. The study validated that HOXA9 promotes the malignant transformation of immortalized astrocytes and induces temozolomide drug resistance via Bcl-2 upregulation. Targeting Bcl-2 with ABT-737 significantly reversed temozolomide resistance in HOXA9-expressing cells [125].

**Fig. 2:**
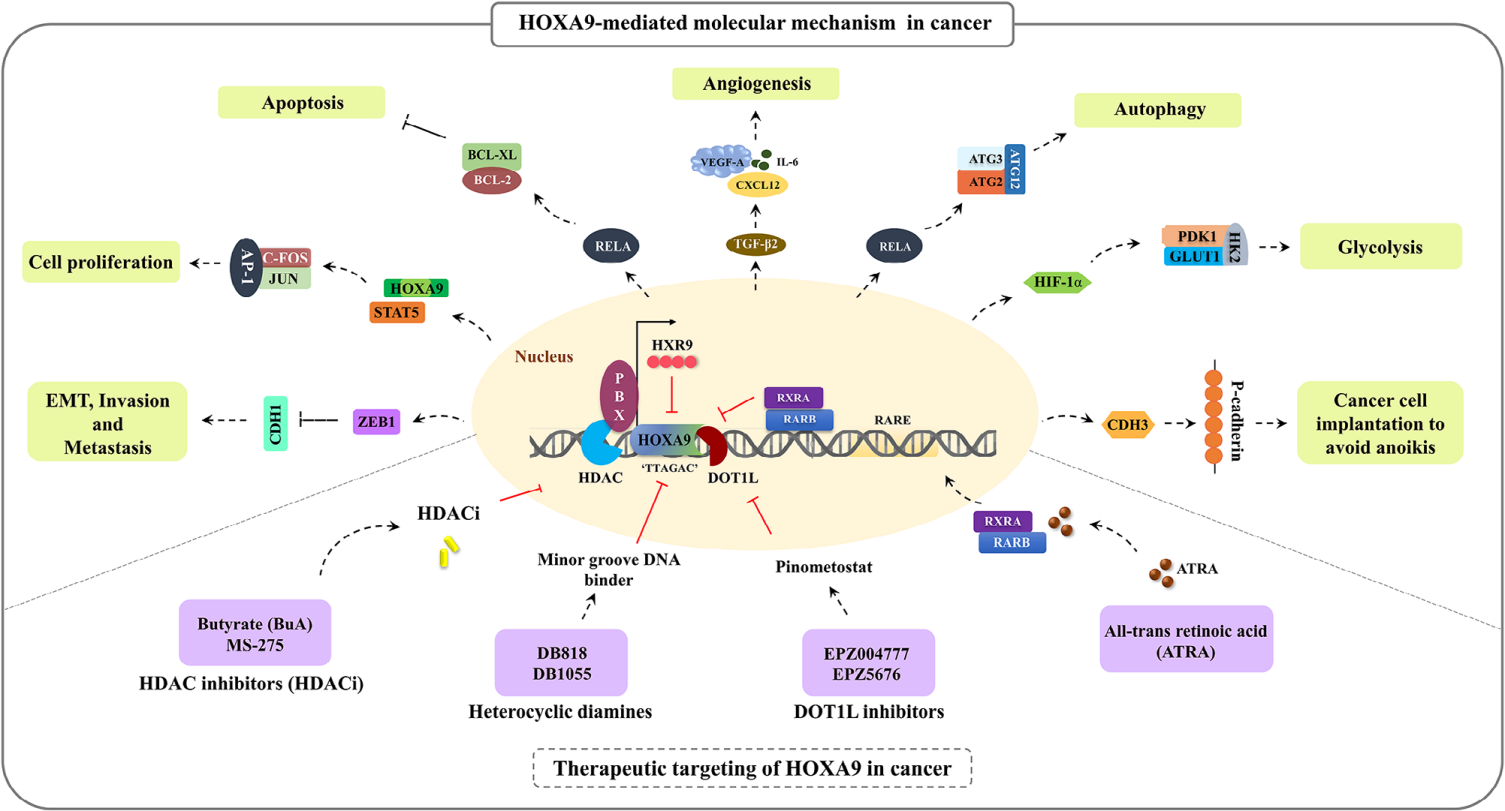
HOXA9-mediated molecular mechanism and its functional implications during cancer progression

Figure 2 summarizes the molecular mechanism and functional implications of HOXA9 during cancer progression.

### Multifunctional role of HOXA9 in cancer

HOX proteins are well-known transcription factors that play a fundamental role in several cellular processes by binding to the promoter region of target genes with their DNA-binding homeodomain [4]. HOXA9 directly targets insulin-like growth factor 1 (*IGF1*) by binding to specific regions on its promoter, including the first intronic and DNase-hypersensitive region. This interaction leads to the stimulation of autocrine signaling, which is needed for hematopoietic transformation [126]. However, dysregulation of HOXA9 often leads to its binding to the promoter of oncogenes, resulting in cancer development. This section provides a comprehensive overview of the extensive role of HOXA9 as a transcription factor under normal physiological conditions and during the progression of cancer (Table 3).

**Table 3:** Role of HOXA9 as a transcription factor

HOXA9 as a transcription factor in normal physiological conditions		
Normal physiological function	HOXA9 targets	References
Hematopoietic cell differentiation	IGF1	[126]
Endothelial cell differentiation	SELE	[176]
Lymphoid and B-cell development	FLT3	[177]
Myeloid differentiation	CYBB	[178]
Endothelial tube formation	EPHB4	[58]
HOXA9 as a transcription factor in cancer		
Cancer	HOXA9 targets	References
Ovarian cancer	TGF-β2, CDH3	[78, 80]
Leukemia	BCL2, SOX4, CDX4, PIM1	[91, 92, 94, 179]
Cervical cancer	GRHL2, IL17RD, FADD, CHEK2, TRAIL, IL-3/IL-5	[19, 76]
Glioma	TRAIL, PTEN, HOTAIR,WNT6	[95, 105, 180]
Breast cancer	BRCA1	[98]
Cutaneous squamous cell carcinoma	HIF-1α, RELA	[87, 90]

Interestingly, Shi et al., 2001 demonstrated that HOXA9 acts as a strong transcriptional repressor in the TGF-β pathway. Smad-4 interacts with HOXA9 and displaces it from its DNA-binding site, thereby facilitating the transcriptional activation of the *OPN* (osteopontin) promoter upon TGF-β stimulation [127].

Furthermore, Ohno et al. (2013) reported the nontranscriptional activity of HOXA9. It was discovered that HOXA9 acts as an E3 ubiquitin ligase in hematopoietic cells, leading to the degradation of the DNA replication inhibitor Geminin through ubiquitination [128]. HOXA9 transduction results in the stimulation of the activity of hematopoietic stem cells (HSCs) and progenitor cells, enhancing their activity [128]. However, its complex function in controlling a wide range of biological phenomena is mostly attributable to the tissue-specific expression of the protein and its collaboration with cofactors and binding partners.

HOXA9 is frequently associated with three-amino loop extension (TALE) homeodomain-containing cofactors, namely, MEIS1 and PBX [129, 130]. HOXA9 cooperates with PBX with a highly conserved motif called hexapeptide (HX), and the additional binding of MEIS induces the remodelling of the trimeric complex. In a study utilizing Bimolecular Fluorescence Complementation (BiFC) in cell lines, researchers discovered that the interaction between HOXA9-TALE protein is dependent on the specific activity of the HX motif and paralogue-specific motif of the HD domain [131]. The association of HOXA9 with PBX3 cofactors has been widely reported in leukemia and in a few solid cancer types [100, 132, 133]. The coexpression and association of HOXA9 with PBX3 enhance their binding to the promoter region of downstream target genes, potentially leading to a worse prognosis in cancer. It has been reported that poor prognosis in GC patients with upregulated HOXA9/PBX3 expression was found to be associated with lymph node metastasis and TNM stage (P value < 0.05) [100]. High coincidental expression and association of HOXA9 and PBX3 have demonstrated a synergistic impact on leukemic cell transformation and leukemogenesis. The study showed a strong association between HOXA9 and PBX3, specifically in patients with different subtypes of AML, including MLL-AML and cytologically abnormal AML (CA-AML) [132, 133]. Researchers have proposed the development of the HXR9 peptide as a potential strategy to disrupt the interaction between HOXA9 and PBX3, aiming to mitigate leukemogenesis [132].

Knockdown of HOXA9/TALE in cytologically normal AML (CN-AML) led to a reduction in aggressiveness and increased sensitivity of cancer cells to cytarabine chemotherapy [134]. In AML, the NPM + mutation has been found to induce high expression of HOXA9-PBX3, resulting in increased di- and trimethylation at the H3K79 locus. The study additionally demonstrated the use of a DOTL1 inhibitor (EPZ5676) to target HOXA9/PBX3, which effectively inhibited the survival of leukemic cells by inducing cell apoptosis [135]. Therefore, TALE factors can be considered potential pathological cofactors of HOXA9 in driving carcinogenesis.

## Clinical applications of HOXA9 in cancer

HOXA9 serves as a potential biomarker for cancer diagnosis and prognosis. Determining the correlation between its expression patterns and disease progression aids clinicians in predicting patient outcomes, ultimately leading to suitable therapeutic interventions. In this section, we have summarized the clinical significance of HOXA9 in different cancer types.

### HOXA9 as a diagnostic and prognostic marker

Accurate and early diagnosis of the condition is crucial for reducing the severity of disease progression and improving prognosis. HOXA9 and its binding partner MEIS1 have been identified as diagnostic markers for early cancer detection in paediatric acute leukaemia. Their expression has been observed to be inversely correlated with overall survival [111]. A recent study has demonstrated the upregulation of HOXA9/MEIS1 in adult acute leukaemia, suggesting its potential as a diagnostic marker for this condition. However, no significant association was observed between disease-free survival (DFS) and overall survival (OS) in the study population [136]. Indeed, several studies have demonstrated that *HOXA9* methylation can serve as a valuable diagnostic and prognostic marker in various types of solid and hematological malignancies. It has also been suggested as a promising therapeutic target for these cancers [9, 69]. Methylation at the *HOXA9* locus has emerged as a widely used biomarker in HNSC [17, 137, 138], BC [18], OVC [16, 112, 113], CC [19], lung cancer [115, 116, 139–141], PCa [75] and bladder cancer [109, 110, 142]. Hence, regular monitoring of *HOXA9* methylation levels can provide valuable insights for making accurate clinical decisions.

### HOXA9 as a marker for clinical staging application

Few studies have reported the emerging role of HOXA9 in differentiating clinical stages, which not only allows the detection of cancer at the early stage but also helps in guiding therapeutic decisions, which eventually aids in better clinical outcomes. In nasopharyngeal cancer (NPC), high expression of HOXA9 has been associated with advanced tumor stage (T), implying its role in cancer progression (P value < 0.05). Multivariate analysis revealed a worse prognosis in patients with HOXA9 overexpression [101]. In addition, HOXA9 overexpression has been significantly associated with higher TNM stage and positive lymph node metastasis in CRC [84].

In contrast, HOXA9 has been found to be downregulated in CC [18]. Downregulation of HOXA9 in CC patients (N = 154) has been significantly associated with clinical outcomes, including TNM stage, pathological grade, and differentiation (P value < 0.05) [143]. Furthermore, increased *HOXA9* methylation levels were observed in more aggressive tumors, and these methylation levels strongly correlated with tumor number, size, grade, and stage in NMIBC [108]. In GC, the upregulation of HOXA9 expression was associated with cell differentiation and clinical staging, particularly in lymph node metastasis [100].

#### Therapeutic targeting of HOXA9 in cancer

Although aberrant expression of HOXA9 has been implicated in many cancer types, pharmacologically targeting HOXA9 in various cancer types presents a significant challenge owing to the complexity of the structure of HOXA9, its interaction with several binding partners, and its involvement in normal physiological processes. Nevertheless, a few studies have proposed the pharmacological targeting of HOXA9 in leukemia. In this section, we will discuss the emerging drugs that have undergone preclinical and clinical trials for the treatment of hematological and solid malignancies. However, the development of suitable drugs and targeted therapies specifically aimed at HOXA9 in solid malignancies is still in the early stages of research.

Lambert et al., 2019 conducted a comprehensive review on various strategies for targeting HOXA9 in AML. The overexpression of HOXA9 can be effectively targeted either by suppressing its expression through epigenetic modulation or by inhibiting its activity at the protein level [144]. Heterocyclic diamines, namely, DB818 and DB1055, were proposed as inhibitors of the HOXA9 transcription factor in leukemia, which function as minor groove DNA ligands on HOXA9, effectively competing with the HOXA9/DNA interaction [145]. Later, it was proposed that DB818 efficiently downregulated the expression of HOXA9 target genes such as the *MYB, MYC, and BCL2* genes in AML [146].

DOT1L, a histone H3-lysine 79 (H3K79) methyltransferase enzyme, plays a crucial role in cell proliferation and cell survival in MLL. It facilitates methylation at the H3K79 position, leading to the overexpression of HOXA9 and MEIS1 [147]. Initially, researchers developed DOT1L inhibitors namely EPZ004777 and EPZ5676, generally known as pinometostat. These inhibitors specifically target and inhibit the activity of the DOT1L enzyme. Treatment with DOT1L inhibitors resulted in the downregulation of HOXA9 and induced cell death in AML cells carrying MLL translocations and NPM1 mutations [135, 148, 149]. Promising results were obtained from successful preclinical studies in xenograft mouse models and phase 1 clinical trials at safer doses, leading to improved clinical outcomes [150–152].

Subsequently, another inhibitor of DOT1L called SYC-522 was developed that shows potential in treating AML. The study revealed that treatment with SYC-522 resulted in a significant decrease in the expression levels of key leukaemia driver genes, including *HOXA9* and *MEIS1*. Furthermore, it also led to a reduction in the expression of cell cycle-related and antiapoptotic genes. SYC-522-mediated inhibition of DOT1L was associated with increased chemosensitivity and improved clinical outcome in AML. Therefore, the combination of a DOT1L inhibitor and chemotherapy holds promise as a reliable approach for the treatment of AML [153]. Several therapeutic drugs, such as LSD1 inhibitors, HDAC class I inhibitors, and WDR5/MLL inhibitors, have demonstrated success in reducing the expression of HOXA9 in leukemia [88, 154–157].

Morgan et al. provided a comprehensive review on the therapeutic targeting of HOXA9 in solid tumors [158]. A synthetic peptide called HXR9 has been developed to specifically target the interaction between HOX and PBX. This peptide has shown promise in reducing the aggressive behavior of cancer cells in a few cancer types [159–161]. In OVC, the disruption of HOX/PBX complexes using the HXR9 peptide has led to HOXA9-mediated transcriptional alteration and induction of apoptosis in cancer cells [161]. In meningioma, targeting HOXA9 with HXR9 has shown promise as a reliable therapeutic option. The synthetic peptide HXR9 has been shown to effectively reduce the proliferation rate of meningioma cells and inhibit tumor aggressiveness. This is achieved by disrupting the HOXA9/PBX interaction, interfering with DNA binding, and subsequently altering the expression of target genes [160]. The HXR9 peptide has demonstrated its effectiveness in inducing apoptosis specifically in premalignant and oral squamous cell carcinoma (OSCC) cells, while having minimal impact on normal oral keratinocytes [162].

There is an enormous gap in targeting the oncogenic nature of HOXA9 in solid tumors. Further investigations are needed to understand the complexity of the regulation of HOXA9 and address off-target effects that may arise when designing and delivering drugs targeting this pathway.

## Conclusion and future prospects

The HOXA9 transcription factor plays a crucial role in various biological processes, including embryonic development, embryo implantation, endothelial cell differentiation, and hematopoiesis. Aberrant expression of HOXA9 triggers it to be oncogenic, leading to the activation of cancer-associated signaling pathways. Its dysregulated expression has been observed in both hematological and solid malignancies, exhibiting context-dependent roles as either an oncogene or tumor suppressor.

This review provides a comprehensive summary of the regulation and functional role of HOXA9 in cancer progression. Several studies have highlighted the significance of HOXA9 as a biomarker for cancer prognosis and clinical staging, emphasizing its versatility in regulating several targets, modulating numerous signaling pathways, and promoting therapy resistance and recurrence. Despite extensive research conducted on HOXA9, there are still some notable research gaps in our understanding of its precise role in cancer.

- Despite being deregulated in various cancer types, the full understanding of HOXA9-mediated molecular events driving cancer progression remains incomplete. Further research is needed to unravel the precise mechanisms through which HOXA9 promotes tumorigenesis and influences disease outcomes in the context of different cancers.
- More studies are required to understand the regulation of HOXA9 by other HOX transcription factors, HOX cluster-embedded lncRNAs and miRNAs. Identifying the binding partners and cofactors of HOXA9 is crucial for unravelling downstream molecular pathways during cancer progression. Gaining a deeper understanding of the functional consequences of HOXA9 dysregulation would provide valuable insights for developing therapeutic interventions.
- Further studies are needed to fully evaluate the clinical significance of HOXA9 in cancer, which is pivotal for the development of targeted therapies.
- Further investigation is essential to investigate the prognostic and diagnostic roles of HOXA9 in larger patient cohorts across various cancer types.
- It is important to explore the feasibility of using HOXA9 as a circulating biomarker in easily accessible bodily fluids such as serum, saliva, plasma, and blood. This approach would not only enhance affordability for patients but also enable clinicians to expedite the diagnosis of disease severity.
- Targeting HOXA9 poses a significant challenge due to its transcriptional complexity, generation of multiple isoforms, tissue-specific expression, involvement in normal physiological functions, and roles in therapy resistance and recurrence. By carefully considering its context-specific dual role, it is crucial to design drugs that specifically and selectively inhibit its activity, minimizing any potential off-target effects.

It is worth noting that understanding the molecular behavior of HOXA9 in cancer requires further investigation. Addressing the existing research gaps is imperative for translating findings into effective therapies.

## Data Availability

Not Applicable. Not applicable.

## Abbreviations

EMT: epithelial-mesenchymal transition
HOX: Homeobox
AML: acute myeloid leukemia
miRNAs: microRNAs
lncRNAs: long noncoding RNAs
CBP: CREB-binding protein
AbdB: Abdominal B
HSC: hematopoietic stem cells
HSPCs: hematopoietic stem and progenitor cells
HEP: hemogenic precursors
EC: Endothelial cell
eNOS: endothelial nitric oxide synthase
VEGFR2: vascular endothelial growth factor receptor 2
CC: cervical cancer
CSCs: cancer stem cells
CRC: colorectal cancer
ATRA: all-trans retinoic acid
EOC: epithelial ovarian cancer
MSCs: mesenchymal stem cells
CAFs: cancer-associated fibroblasts
OVC: ovarian cancer
CSCC: cutaneous squamous cell carcinoma
HDAC: histone deacetylases
PCa: prostate cancer
GBM: glioblastoma
TRAIL: tumor necrosis factor-related apoptosis-including ligand
BC: breast cancer
GC: gastric cancer
TNM: tumor-node-metastasis
NSCLC: non-small cell lung carcinoma
NMIBC: nonmuscle invasive bladder cancer
ctDNA: circulating tumor-specific DNA
MTD: maximum tolerated dose
IGF1: Insulin-like growth factor 1
HX: hexapeptide
TALE: three-amino loop extension
BiFC: Bimolecular Fluorescence Complementation
HGSOC: high-grade serous ovarian cancer
NAC1: nucleus accumbens‑associated protein 1
CA: AML-cytologically abnormal-AML
CN: AML-cytologically normal-AML
PML: paediatric acute leukaemia
OS: overall survival
DFS: disease-free survival
NPC: nasopharyngeal cancer
